# Sidedness and TP53 mutations impact OS in anti-EGFR but not anti-VEGF treated mCRC - an analysis of the KRAS registry of the AGMT (Arbeitsgemeinschaft Medikamentöse Tumortherapie)

**DOI:** 10.1186/s12885-017-3955-4

**Published:** 2018-01-03

**Authors:** Florian Huemer, Josef Thaler, Gudrun Piringer, Hubert Hackl, Lisa Pleyer, Clemens Hufnagl, Lukas Weiss, Richard Greil

**Affiliations:** 1 IIIrd Medical Department with Haematology, Medical Oncology, Haemostaseology, Infectious Diseases and Rheumatology, Oncologic Center, Paracelsus Medical University, 5020 Salzburg, Austria; 2 Salzburg Cancer Research Institute with Laboratory of Immunological and Molecular Cancer Research and Center for Clinical Cancer and Immunology Trials, 5020 Salzburg, Austria; 3 Cancer Cluster Salzburg, 5020 Salzburg, Austria; 4 Department of Internal Medicine IV, Klinikum Wels-Grieskirchen, 4600 Wels, Austria; 5 Division of Bioinformatics, Biocenter, Medical University of Innsbruck, 6020 Innsbruck, Austria

**Keywords:** Colorectal cancer, Sidedness, Anti-VEGF, Anti-EGFR, Bevacizumab, Cetuximab, Panitumumab, TP53, KRAS, Predictive value

## Abstract

**Background:**

In metastatic colorectal cancer (mCRC), the localization of the primary tumour has been shown to be of prognostic as well as predictive relevance.

**Methods:**

With the aim to investigate clinical and molecular disease characteristics with respect to sidedness in a real-world cohort, we analyzed 161 mCRC patients included in the KRAS Registry of the Arbeitsgemeinschaft Medikamentöse Tumortherapie (AGMT) between January 2006 and October 2013.

**Results:**

Right-sided mCRC displayed a worse median overall survival (OS) in comparison to left-sided disease (18.1 months [95%-CI: 14.3–40.7] versus 32.3 months [95%-CI: 25.5–38.6]; HR: 1.63 [95%-CI: 1.13–2.84]; *p* = 0.013). The choice of the biological agent in front-line therapy had a statistically significant impact on median OS in patients with right-sided tumours (anti-epidermal growth factor receptor (EGFR): 10.6 months [95%-CI: 5.2-NA]; anti-vascular endothelial growth factor (VEGF): 26.2 months [95%-CI: 17.9-NA]; HR: 2.69 [95%-CI: 1.30–12.28]; *p* = 0.015) but not in patients with left-sided tumours (anti-EGFR: 37.0 months [95%-CI: 20.2–56.6]; anti-VEGF: 32.3 months [95%-CI: 23.6–41.1]; HR: 0.97 [95%-CI: 0.56–1.66]; *p* = 0.905). When evaluating molecular characteristics of tumour samples, we found a clinically meaningful trend towards an inferior OS in TP53 mutant mCRC treated with anti-EGFR based therapy compared to anti-VEGF based therapy (17.1 months [95%-CI: 8.7-NA] versus 38.3 months [95%-CI: 23.6–48.0], HR = 1.95 [95%-CI: 0.95–5.88]; *p* = 0.066), which was not significantly dependent on sidedness. This was not the case in patients with TP53 wild-type tumours. Therefore we evaluated the combined impact of sidedness and TP53 mutation status in the anti-EGFR treated cohort and patients with left-sided/TP53 wild-type mCRC showed the longest median OS (38.9 months) of all groups (right-sided/TP53 mutant: 12.1 months; right-sided/TP53 wild-type: 8.9 months; left-sided/TP53 mutant: 18.4 months; *p* = 0.020).

**Conclusions:**

TP53 mutation and right-sidedness are associated with shorter OS in patients treated with anti-EGFR based therapy but not with anti-VEGF based therapy. The confirmation of the predictive value of TP53 mutation status in a larger cohort is warranted.

**Electronic supplementary material:**

The online version of this article (10.1186/s12885-017-3955-4) contains supplementary material, which is available to authorized users.

## Background

Colorectal cancer accounts for 13% of all new cancer cases diagnosed each year and is the second leading cause of cancer-related death in Europe [1]. One fifth of patients present with distant metastases at initial diagnosis and the treatment approach for most patients with metastatic colorectal cancer (mCRC) is palliative [2]. Mounting evidence suggests that the localization of the primary tumour may impact clinical behaviour of mCRC [3]. While the right-sided colon (from the appendix to the right-lateral two-thirds of the transverse colon) develops from the embryonic midgut, the left colon (from the left-lateral one-third of the transverse colon to the rectum) derives from the hindgut. Right-sided tumours more often exhibit BRAF-mutations, microsatellite instability, CpG island methylator phenotype, mucinous differentiation and serrated pathway signature. In contrast, left-sided tumours more often show chromosomal instability and amplification of the epidermal growth factor receptor (EGFR) or human epidermal growth factor receptor 2 and epiregulin tends to be overexpressed [3–5]. There is a negative gradient of infiltrating immune cells from the right to the left colon with significantly increased immune activity in the healthy adult caecum compared to the rectum [6]. Furthermore, the microbial load as well as the development of biofilms along the colorectal axis, which may also impact on local immunocompetence, distinguishes right-sided from left-sided colorectal cancer [7].

Patients with mCRC originating from right-sided tumours are reported to display a worse overall survival (OS) compared to left-sided tumours and retrospective analyses of the CALGB-80405 and FIRE-3 studies demonstrated a predictive value of the primary tumour localization and the choice between anti-vascular endothelial growth factor (VEGF) and anti-EGFR based systemic therapy in mCRC [2, 8–10]. A retrospective analysis of the US-American CALGB-80405 trial demonstrated a statistically significant difference in OS between patients treated with anti-EGFR based and anti-VEGF based therapy in left-sided mCRC (36.0 versus 31.4 months; HR: 0.82; *p* = 0.01) but not in right-sided tumours (16.7 versus 24.2 months; HR: 1.26; *p* = 0.08, 9]. Similarly, a retrospective analysis of the European FIRE-3 trial could show a pronounced difference in median OS in favour of anti-EGFR based therapy in left-sided mCRC (28.0 versus 38.3 months; HR: 0.63; *p* = 0.002), but not in right-sided disease (18.3 versus 23.0 months; HR: 1.44; *p* = 0.28,) [8]. The biologic basis for the worse outcome with anti-EGFR based therapy in right-sided tumours is so far unknown and even classification of tumours according to the Consensus Molecular Subtypes (CMS) could not clarify this issue [3, 11, 12].

In consideration of these results, we aimed at investigating the prognostic and predictive value of primary tumour localization in our bicentric real-world cohort of 161 mCRC patients outside of a clinical trial. Furthermore, the distribution of molecular alterations and baseline clinical characteristics were studied.

## Methods

This retrospective analysis of the KRAS Registry of the Arbeitsgemeinschaft Medikamentöse Tumortherapie (AGMT) was approved by the Ethics Committee of the provincial government of Salzburg, Austria (Nr. 1146) and was based on the data of 161 unselected consecutive patients with mCRC diagnosed and/or treated at the tertiary cancer centres in Salzburg or Wels, Austria between January 2006 and October 2013. The KRAS Registry is a non-interventional, retrospective and prospective, multi-centre research initiative investigating the standards of KRAS testing and clinical outcome in mCRC. Systemic therapy was applied according to local and international standards. All patients included in the registry signed an informed consent. OS was calculated from the date of first diagnosis of metastatic disease until date of death or date of last known follow-up. The categorization of primary tumour localization was performed according to previous reports [3, 8]. Genomic DNA was extracted from paraffin-embedded primary tumour samples using the Maxwell DNA LEV tissue DNA kit (Promega, WI, USA). Following PCR-amplification genes of interest were sequenced using the capillary sequencer ABI 3100 Analyser (Applied Biosystems, CA, USA). Mutational analyses included KRAS (exons 2–4), NRAS (exons 2–4), TP53 (exons 5–9), BRAF (exon 15) and phosphatidylinositol-3-kinase (PI3K; exons 9 and 20). For primers and probes see Additional file 1: Table S1. Extended RAS mutational status summarizes mutations in KRAS and NRAS. Anti-VEGF antibodies included bevacizumab and aflibercept, anti-EGFR antibodies included cetuximab and panitumumab. Anti-EGFR based front-line therapy was restricted to extended RAS wild-type patients.

Differences in patient baseline characteristics and molecular alterations between left-sided and right-sided mCRC were tested by Pearson’s χ^2^-test with Yates’ correction or for small number of expected counts (E⩽5) by two-sided Fisher’s exact test as indicated. Where stated the differences between left-sided and right-sided mCRC were based on the number of patients in individual categories compared to the remaining patients in the respective group. For continuous data the difference between the two groups were calculated with two-sided Wilcoxon rank-sum test. Survival curves were estimated by the Kaplan–Meier method. Log-rank test (corresponding to a two-sided Z-test) was used to compare survival distributions between two (or where indicated four) patient groups and is considered appropriate for censored survival data analysis. Multivariate Cox regression analyses on overall survival were performed stratified according to therapy and included sidedness, TP53 mutation status and their interaction as covariates. *P*-values were adjusted for multiple testing based on the false discovery rate according to the Benjamini-Hochberg method. Proportional hazard assumptions were tested and not violated. All analyses were performed using the statistical environment R (version 3.3.1, Austria) including package survival.

## Results

### Baseline characteristics and sidedness

Baseline characteristics are depicted in Table 1. Among the 161 patients included in our registry, 76% had left-sided and 24% had right-sided tumours. In 63 patients (39%) the primary tumour originated from the rectum. The distribution between synchronously and metachronously metastasized disease did not differ by side (X^2^-test *p* = 0.427). A higher frequency of mucinous differentiation in tumours originating in the right than in the left colon was observed (21% versus 8%, χ^2^-test *p* = 0.038). Lung metastases were more frequently associated with left-sided mCRC (36% versus 18%, χ^2^-test *p* = 0.070). The number of liver-limited disease was equally distributed between sides (right-sided: 37% versus left-sided: 37%, χ^2^-test *p* = 1.000) as were concurrent hepatic and peritoneal metastases (right-sided: 11%; left-sided: 9%; Fisher’s exact test *p* = 0.754). Eleven patients (7%) received best supportive care only. Of the remaining 150 patients receiving systemic therapy, 41 patients (25%) were treated with chemotherapy alone in first-line, anti-VEGF based (53% versus 48%, χ^2^-test *p* = 0.751) and anti-EGFR based (21% versus 18%, χ^2^-test *p* = 0.781) systemic front-line therapy was equally distributed between right-sided and left-sided mCRC. The choice of the chemotherapy backbone for first-line systemic therapy did not significantly differ between sides. Metastasectomy with curative intent was performed in 13% of patients with right-sided mCRC as compared to 25% with left-sided mCRC (χ^2^-test *p* = 0.197).

**Table 1:** Distribution of baseline characteristics between right-sided and left-sided metastatic colorectal cancer among 161 patients

	All (*n* = 161)	Right-sided mCRC (*n* = 38)	Left-sided mCRC (*n* = 123)	*p*-value
Sex				
Male^a^	103 (64)	26 (68)	77 (63)	0.646
Female^a^	58 (36)	12 (32)	46 (37)	
Median age at diagnosis of metastatic disease				
(range)^a^	65 (35–85)	67.5 (35–85)	65 (39–84)	0.127^d^
Grading	161 (100)			
I	1 (1)	0 (0)	1 (1)	1.000
II^a^	102 (63)	25 (66)	77 (63)	
III^a^	51 (32)	13 (34)	38 (31)	
Not available	7	0	7	
Detection of metastases				
Synchronous^a^	108 (67)	28 (74)	80 (65)	0.427
Metachronous^a^	53 (33)	10 (26)	43 (35)	
Histologic subtype				
Non-mucinous^a^	143 (89)	30 (79)	113 (92)	0.038^c^
Mucinous^a^	18 (11)	8 (21)	10 (8)	
Location of first metastases				
Liver^b,e^	108 (67)	28 (74)	80 (65)	0.427
Lung^b,e^	51 (32)	7 (18)	44 (36)	0.070
Peritoneum^b,e^	31 (19)	9 (24)	22 (18)	0.578
Other^b,e^	38 (24)	11 (29)	27 (22)	0.503
Liver and peritoneum^b,e^	15 (9)	4 (11)	11 (9)	0.754^c^
Liver-limited metastases				
Yes^a^	59 (37)	14 (37)	45 (37)	1.000
No^a^	102 (63)	24 (63)	78 (63)	
First-line systemic therapy				
Anti-VEGF based^b^	79 (49)	20 (53)	59 (48)	0.751
Bevacizumab	76	20	56	
Aflibercept	3	0	3	
Anti-EGFR based^b^	30 (19)	8 (21)	22 (18)	0.781
Cetuximab	20	5	15	
Panitumumab	10	3	7	
Chemotherapy only^b^	41 (25)	9 (24)	32 (26)	0.940
No systemic therapy	11 (7)	1 (2)	10 (8)	
Metastasectomy with curative intent				
Yes^a^	36 (22)	5 (13)	31 (25)	0.197
No^a^	123 (77)	32 (84)	91 (74)	
Not available	2 (1)	1 (3)	1 (1)	
Chemotherapy backbone				
Oxaliplatin^a^	91 (56)	24 (63)	67 (55)	0.712
Irinotecan^a^	40 (25)	9 (23)	31 (25)	
5-FU/Capecitabine mono^a^	19 (12)	4 (11)	15 (12)	
No Chemotherapy^a^	11 (7)	1 (3)	10 (8)	

Percentage in brackets, ^a^included categories, ^b^number of patients in individual categories versus all other patients in the respective group, ^c^two-sided Fisher’s exact test, ^d^two-sided Wilcoxon rank-sum test, ^e^multiple designations are possible, Χ^2^-test with Yates’ correction in all other cases

### Molecular characterization and sidedness

**Fig. 1:**
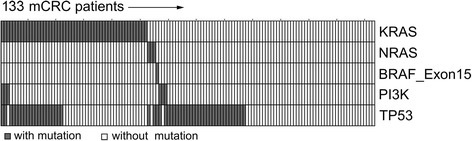
Heat map of molecular alterations among 133 metastatic colorectal cancer patients. In 28 patients included in the KRAS Registry of the Arbeitsgemeinschaft Medikamentöse Tumortherapie (AGMT), at least one molecular analysis of KRAS, NRAS, BRAF, PI3K and/or TP53 was missing, therefore these patients were excluded from the illustration

Results of the molecular analyses are shown in Table 2. Extended RAS analysis was available in 154 patients and RAS mutations were detected in 65 patients (42%). The frequency of RAS mutations did not differ by side (right-sided: 50% versus left-sided: 40%, χ^2^-test *p* = 0.352). TP53 mutations were more frequent in left-sided than right-sided mCRC (47% versus 22%, χ^2^-test *p* = 0.012). The distribution of BRAF mutations and PI3K mutations did not significantly differ between sides. KRAS, NRAS and BRAF mutations were mutually exclusive as depicted in Fig. 1.

**Table 2:** Distribution of molecular alterations between right-sided and left-sided metastatic colorectal cancer

		All (*n* = 161)	Right-sided mCRC (*n* = 38)	Left-sided mCRC (*n* = 123)	*p*-value
Extended RAS status	Wild-type^a^	89 (58)	19 (50)	70 (60)	0.352^b^
	Mutant^a^	65 (42)	19 (50)	46 (40)	
	Not available	7	0	7	
KRAS Exon 2	Wild-type^a^	97 (62)	20 (53)	77 (65)	0.229^b^
	Mutant^a^	59 (38)	18 (47)	41 (35)	
	Not available	5	0	5	
KRAS Exon 3	Wild-type^a^	145 (99)	38 (100)	107 (99)	1.000^b^
	Mutant^a^	1 (1)	0 (0)	1 (1)	
	Not available	15	0	15	
KRAS Exon 4	Wild-type^a^	144 (98)	37 (97)	107 (98)	1.000^b^
	Mutant^a^	3 (2)	1 (3)	2 (2)	
	Not available	14	0	14	
NRAS Exon 2	Wild-type^a^	144 (99)	38 (100)	106 (98)	1.000^b^
	Mutant^a^	2 (1)	0 (0)	2 (2)	
	Not available	15	0	15	
NRAS Exon 3	Wild-type^a^	145 (99)	38 (100)	107 (99)	1.000^b^
	Mutant^a^	1 (1)	0 (0)	1 (1)	
	Not available	15	0	15	
NRAS Exon 4	Wild-type^a^	146 (100)	38 (100)	108 (100)	1.000^b^
	Mutant^a^	0 (0)	0 (0)	0 (0)	
	Not available	15	0	15	
TP53 mutation	Wild-type^a^	84 (60)	29 (78)	55 (53)	0.012^b^
	Mutant^a^	57 (40)	8 (22)	49 (47)	
	Not available	20	1	19	
TP53 Exon 5	Wild-type^a^	113 (80)	32 (86)	81 (78)	0.375^b^
	Mutant^a^	28 (20)	5 (14)	23 (22)	
	Not available	20	1	19	
TP53 Exon 6	Wild-type^a^	135 (96)	36 (97)	99 (95)	1.000^b^
	Mutant^a^	6 (4)	1 (3)	5 (5)	
	Not available	20	1	19	
TP53 Exon 7	Wild-type^a^	131 (93)	35 (95)	96 (92)	0.728^b^
	Mutant^a^	10 (7)	2 (5)	8 (8)	
	Not available	20	1	19	
TP53 Exon 8	Wild-type^a^	126 (89)	37 (100)	89 (86)	0.012^b^
	Mutant^a^	15 (11)	0 (0)	15 (14)	
	Not available	20	1	19	
TP53 Exon 9	Wild-type^a^	86 (100)	25 (100)	61 (100)	NA
	Mutant^a^	0 (0)	0 (0)	0 (0)	
	Not available	75	13	62	
BRAF Exon 15	Wild-type^a^	136 (99)	34 (100)	102 (99)	1.000^b^
	Mutant^a^	1 (1)	0 (0)	1 (1)	
	Not available	24	4	20	
PI3K	Wild-type^a^	129 (96)	34 (97)	95 (95)	0.677^b^
	Mutant^a^	6 (4)	1 (3)	5 (5)	
	Not available	26	3	23	
PI3K Exon 9	Wild-type^a^	132 (99)	34 (100)	98 (98)	1.000^b^
	Mutant^a^	2 (1)	0 (0)	2 (2)	
	Not available	27	4	23	
PI3K Exon 20	Wild-type^a^	137 (97)	36 (97)	101 (97)	1.000^b^
	Mutant^a^	4 (3)	1 (3)	3 (3)	
	Not available	20	1	19	

Percentage in brackets, ^a^included categories, ^b^two-sided Fisher’s exact test, Χ^2^-test with Yates’ correction in all other cases

### Clinical outcome and sidedness

#### Prognostic value

We observed a significant association with shorter OS in right-sided when compared to left-sided mCRC (median OS: 18.1 months [95%-CI: 14.3–40.7] versus 32.3 [95%-CI: 25.5–38.6] months; HR: 1.63 [95%-CI: 1.13–2.84]; *p* = 0.013). RAS mutations did not significantly impact on median OS in the entire cohort (mutant: 27.3 months [95%-CI: 23.1–38.2]; wild-type: 28.0 months [95%-CI: 21.4–38.9]; HR: 1.12 [95%-CI: 0.78–1.62]; *p* = 0.536). TP53 mutations were not significantly associated with shorter median OS compared to TP53 wild-type tumours (24.1 months [95%-CI: 19.2–38.4] versus 28.0 [95%-CI: 22.7–38.9] months; HR: 1.22 [95%-CI: 0.84–1.78]; *p* = 0.289). Mutations in the PI3K gene did not impact on median OS in comparison to PI3K wild-type disease (17.5 months [95% CI: 8.7-NA] versus 27.3 [95% CI: 23.1–37.8]; HR = 1.38 [95% CI: 0.56–3.88]; *p* = 0.430).

In order to detect a possible statistical interaction between sidedness and TP53 mutation status we performed multivariate Cox-regression analysis: after stratification according to therapy sidedness showed a negative impact on OS (HR: 1.77 [95%-CI: 1.06–2.95]; *p* = 0.030) whereas this was not the case for TP53 mutations (HR: 1.47 [95%-CI: 0.93–2.30]; *p* = 0.097; Table 3). Median OS was significantly longer in patients who had undergone metastasectomy with curative intent in comparison to patients that only received palliative systemic therapy (median OS: 55.2 months [95%-CI: 44.9-NA] versus 23.1 months [95%-CI: 18.2–27.3]; HR: 0.31 [95%-CI: 0.27–0.56]; *p* < 0.001).

**Table 3:** Multivariate overall survival analyses including sidedness, the TP53 mutation status, and their interaction as covariates

	Coeff	e^Coeff^ (HR)	95%-CI	*p*	adjusted p
Stratified according to therapy (*n* = 141, number of events = 118)					
Sidedness	0.57	1.77	1.06–2.95	0.030	0.090
TP53 mutation status	0.38	1.47	0.93–2.30	0.097	0.145
Sidedness: TP53 mutation status interaction	0.04	1.04	0.40–2.74	0.930	0.930
Anti-VEGF therapy (*n* = 72, number of events = 60)					
Sidedness	0.34	1.40	0.71–2.76	0.326	0.489
TP53 mutation status	0.21	1.23	0.67–2.26	0.507	0.507
Sidedness: TP53 mutation status interaction	−1.20	0.30	0.04–2.52	0.268	0.489
Anti-EGFR therapy (*n* = 29, number of events = 26)					
Sidedness	1.29	3.64	1.27–10.4	0.016	0.049
TP53 mutation status	1.00	2.71	1.02–7.17	0.045	0.068
Sidedness: TP53 mutation status interaction	−0.39	0.67	0.10–4.55	0.686	0.686

Multivariate survival analysis using Cox’s regression model - stratified according to therapy, for the group of anti-VEGF treated patients, and for the group of anti-EGFR treated patients

### Predictive value

**Fig. 2:**
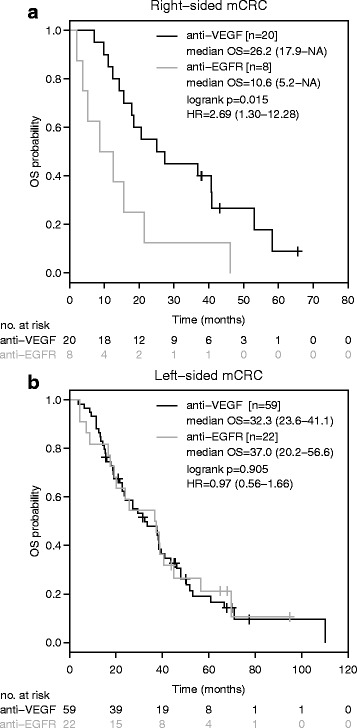
Overall survival according to anti-EGFR/anti-VEGF based therapy and sidedness in metastatic colorectal cancer. Kaplan-Meier curves for overall survival in right-sided (**a**) and left-sided (**b**) mCRC patients receiving anti-EGFR based or anti-VEGF based front-line therapy. HR is hazard ratio, 95% confidence interval in brackets

**Fig. 3:**
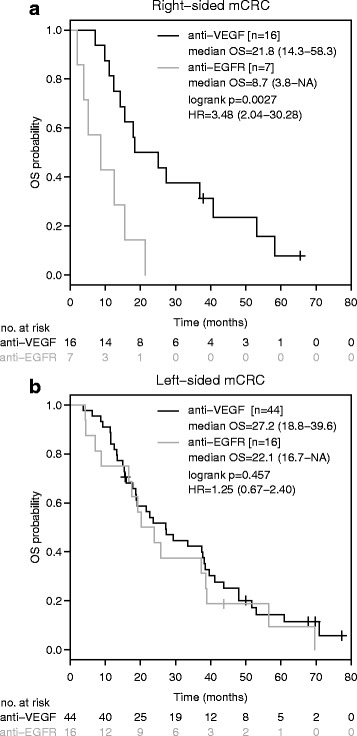
Overall survival according to anti-EGFR/anti-VEGF based therapy and sidedness in patients without potentially curative metastasectomy. Kaplan-Meier curves for overall survival in right-sided (**a**) and left-sided (**b**) mCRC patients receiving anti-EGFR based or anti-VEGF based front-line therapy, excluding patients who had undergone potentially curative metastasectomy. HR is hazard ratio, 95% confidence interval in brackets

Median OS among patients with right-sided mCRC was significantly shorter with front-line anti-EGFR based therapy in contrast to anti-VEGF based therapy (anti-EGFR: 10.6 months (95%-CI: 5.2-NA); anti-VEGF: 26.2 months [95%-CI: 17.9-NA]; HR: 2.69 [95%-CI: 1.30–12.28]; *p* = 0.015, Fig. 2a). In contrast, no difference in median OS was observed between anti-EGFR and anti-VEGF based front-line therapy in patients with left-sided disease (37.0 months [95%-CI: 20.2.-56.6] versus 32.3 months [95%-CI: 23.6–41.1]; HR: 0.97 [95%-CI: 0.56–1.66]; *p* = 0.905, Fig. 2b). We could corroborate this finding even after exclusion of patients who had undergone metastasectomy with curative intent, although OS was considerably shorter:

median OS with right-sided tumours was inferior with first-line anti-EGFR based therapy in comparison to anti-VEGF based therapy (anti-EGFR: 8.7 months [95%-CI: 3.8-NA]; anti-VEGF: 21.8 months [95%-CI: 14.3–58.3]; HR: 3.48 [95%-CI: 2.04–30.28]; *p* = 0.0027, Fig. 3a) while no difference was shown with left-sided disease (anti-EGFR: 22.1 months [95%-CI: 16.7-NA]; anti-VEGF: 27.2 months [95%-CI: 18.8–39.6]; HR: 1.25 [95%-CI: 0.67–2.40]; *p* = 0.457; Fig. 3b).

**Fig. 4:**
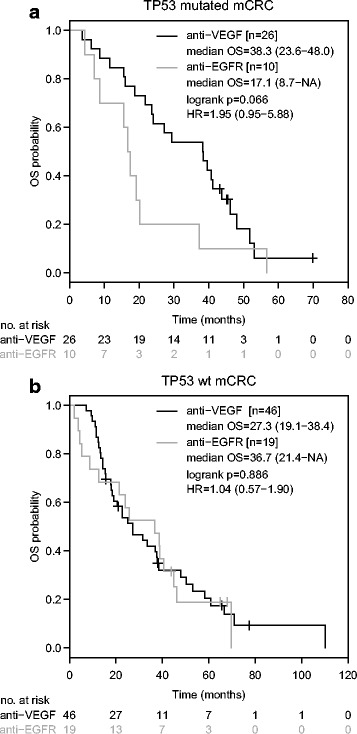
Overall survival according to anti-EGFR/anti-VEGF based therapy and TP53 mutation status in metastatic colorectal cancer. Kaplan-Meier curves for overall survival in TP53 mutant (**a**) or TP53 wild-type (**b**) disease with first-line anti-EGFR or anti-VEGF based therapy. HR is hazard ratio, 95% confidence interval in brackets

**Fig. 5:**
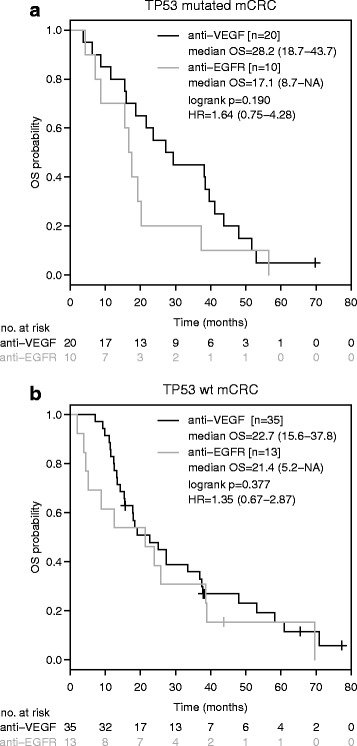
Overall survival according to anti-EGFR/anti-VEGF based therapy and TP53 mutation status in metastatic colorectal cancer. Kaplan-Meier curves for overall survival in TP53 mutant (**a**) or TP53 wild-type (**b**) disease with first-line anti-EGFR or anti-VEGF based therapy, excluding patients who had undergone potentially curative metastasectomy. HR is hazard ratio, 95% confidence interval in brackets

A trend towards shorter OS was observed in patients with TP53 mutated disease who had been treated with anti-EGFR based first-line therapy compared to anti-VEGF based therapy (median OS 17.1 months [95%-CI: 8.7-NA] versus 38.3 months [95%-CI: 23.6–48.0]; HR: 1.95 [95%-CI: 0.95–5.88]; *p* = 0.066, Fig. 4a). In contrast, the choice of the biological agent did not impact median OS in TP53 wild-type tumours (anti-EGFR: 36.7 months [95%-CI: 21.4-NA]; anti-VEGF: 27.3 months [19.1–38.4]; HR: 1.04 [95%-CI: 0.57–1.90]; *p* = 0.886; Fig. 4b). After exclusion of patients who had undergone metastasectomy with curative intent, a numerical difference in median OS in favour of anti-VEGF based front-line therapy was observed (anti-EGFR: 17.1 months [95%-CI: 8.7-NA]; anti-VEGF: 28.2 months [95%-CI: 18.7–43.7]; HR = 1.64 [95%-CI: 0.75–4.28]; *p* = 0.190; Fig. 5a) while TP53 wild-type disease did not favour any biological agent (anti-EGFR: 21.4 months [95-% CI: 5.2-NA]; anti-VEGF: 22.7 months [95%-CI: 15.6–37.8]; HR: 1.35 [95%-CI: 0.67–2.87]; *p* = 0.377; Fig. 5b).

Of interest, in the group of anti-VEGF treated patients, multivariate analysis including sidedness and TP53 mutation status did not show a significant impact of these factors on OS. However when analyzing anti-EGFR treated patients, multivariate analysis including sidedness and TP53 mutation status showed a significant impact of both factors on OS (TP53 mutation: HR: 2.71 [95%-CI: 1.02–7.17]; *p* = 0.045); sidedness: HR: 3.64 [95%-CI: 1.27–10.4]; *p* = 0.016) which were not significantly dependent on each other (Table 3).

**Fig. 6:**
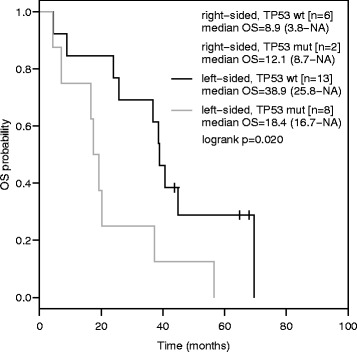
Overall survival according to sidedness and TP53 mutation status in first-line anti-EGFR treated metastatic colorectal cancer. Kaplan-Meier curves for overall survival in right-sided/TP53 mutant, right-sided/TP53. wild-type, left-sided/TP53 mutant and left-sided/TP53 wild-type metastatic colorectal cancer

Furthermore, we evaluated the combined impact of sidedness and TP53 mutation status on OS in mCRC patients treated with first-line anti-EGFR based therapy by creating four groups:

1) right-sided/TP53 mutant mCRC, 2) right-sided/TP53 wild-type mCRC, 3) left-sided/TP53 mutant mCRC and 4) left-sided/TP53 wild-type mCRC. Median OS for these groups was 12.1, 8.9, 18.4 and 38.9 months (*p* = 0.020, Fig. 6).

## Discussion

Primary tumour localization has increasingly come into the focus of mCRC research and is thought to represent a major determinator for clinical management. Differences in pathogenesis, molecular pathways and outcome depending on sidedness have been extensively studied [2–4]. Recent results of retrospective analyses of the CALGB-80405 and FIRE-3 trials demonstrate a benefit in OS with anti-EGFR based front-line therapy in left-sided mCRC in comparison to anti-VEGF based therapy [8, 9] while no statistically significant difference in OS could be detected in right-sided mCRC. However, a retrospective analysis of the PEAK trial only revealed a numerically improved OS with anti-EGFR based therapy in left-sided mCRC when compared to anti-VEGF based therapy without reaching statistical significance [13].

The results of our retrospective analysis of 161 mCRC patients demonstrate a statistically significant survival disadvantage with anti-EGFR based front-line therapy compared to anti-VEGF based therapy in right-sided mCRC (Fig. 2a). This difference in OS prevailed even after excluding patients who had undergone metastasectomy with curative intent (Fig. 3a). We could not detect the superiority of an anti-EGFR based front-line therapy over an anti-VEGF based therapy in left-sided mCRC (Fig. 2b and Fig. 3b), a fact that might be explained by the limited number of included patients.

In our cohort we could confirm a higher frequency of TP53 mutations in left-sided mCRC [14, 15]. Retrospective data from a phase III trial comparing chemotherapy with either bevacizumab or placebo as first-line treatment in mCRC did neither show a prognostic value for TP53 mutation in mCRC, nor a predictive value for the response to bevacizumab based therapy [15]. There is conflicting data on the role of TP53 mutation as a predictive biomarker for anti-EGFR based therapy: in two studies with chemorefractory RAS-unselected or KRAS/BRAF wild-type mCRC patients treated with cetuximab based chemotherapy, TP53 mutation appeared to predict cetuximab sensitivity, particularly in patients with KRAS/BRAF wild-type tumours [16, 17]. In contrast, the phase II trial TEGAFOX-E evaluating the activity of cetuximab in combination with oxaliplatin-based chemotherapy as front-line therapy in RAS-unselected mCRC, did not show a statistically significant difference between TP53 wild-type and TP53 mutant tumours in terms of response rate, progression-free survival or OS [18]. Several other studies did not observe an association between TP53 mutation status and treatment response to cetuximab based therapy in mCRC [19–22]. However, the biomarker analysis of the EXPERT-C trial suggested an OS benefit by adding cetuximab to neoadjuvant chemotherapy in localized rectal cancer patients only with TP53 wild-type tumours [23].

Folprecht et al. reported a higher frequency of PI3K mutations (25.5% versus 14.1%) and BRAF mutations (22.6% versus 5.1%) in right-sided advanced colorectal cancer compared to left-sided disease [24]. In our cohort, PI3K mutations (3.7%) and BRAF mutations (0.6%) were rarely observed. As a consequence, no difference in distribution across sides was detected and therefore correlative studies with clinical parameters have not been performed.

Our analysis revealed a clinically meaningful survival advantage with anti-VEGF based front-line therapy compared to anti-EGFR based therapy in TP53 mutant disease. Despite the limited number of patients, the OS benefit gained by choosing an anti-EGFR based therapy in left-sided mCRC could not be observed in TP53 mutated disease with a median OS comparable to right-sided mCRC (Fig. 6). In line with our results, in vitro data and xenograft models demonstrate a key role of TP53 mutations in acquired resistance to EGFR inhibitors [25, 26].

## Conclusions

In summary, this retrospective analysis of a bicentric real-world cohort of 161 mCRC patients showed a statistically significant OS benefit of front-line anti-VEGF based therapy over anti-EGFR based therapy in right-sided mCRC. Molecular analyses revealed a higher frequency of TP53 mutations in left-sided mCRC. Furthermore, we observed a trend towards superior OS with anti-VEGF based therapy compared to anti-EGFR based therapy in TP53 mutant disease, while there was no difference in TP53 wild-type tumours. Although the patient number was limited, the benefit of first-line anti-EGFR based therapy in left-sided mCRC could not be observed in TP53 mutant disease. If confirmed in a larger cohort, these data might warrant stratification according to sidedness and TP53 mutation status in future mCRC trials investigating anti-EGFR and/or anti-VEGF based systemic therapy.

## Additional file

Additional file 1: Table S1. Primers and probes used for molecular analyses of tumour samples. (DOC 38 kb)

## Abbreviations

AGMT: Arbeitsgemeinschaft Medikamentöse Tumortherapie
CRC: colorectal cancer
EGFR: epidermal growth factor receptor
mCRC: metastatic colorectal cancer
OS: overall survival
PI3K: phosphatidylinositol-3-kinase
VEGF: vascular endothelial growth factor
