# Supplementary material for: Sidedness and TP53 mutations impact OS in anti-EGFR but not anti-VEGF treated mCRC - an analysis of the KRAS registry of the AGMT (Arbeitsgemeinschaft Medikamentöse Tumortherapie)
Source: BMC Cancer. 2018 Jan 3;18:11. doi: 10.1186/s12885-017-3955-4 (PMC5753540; doi:10.1186/s12885-017-3955-4)
Supplement: Additional file 1: Table S1. — Primers and probes used for molecular analyses of tumour samples. (DOC 38 kb) [file 12885_2017_3955_MOESM1_ESM.doc]

**Additional file 1: Table S1.** **Primers and probes used for molecular analyses of tumour samples**

|  | **sequence 5`- 3`** |
| --- | --- |
| TP53_Exon5+6 FOR | ACGCCAACTCTCTCTAGCTCGC |
| TP53_Exon5+6 REV | GGAGGGCCACTGACAACCAC |
| TP53_Exon7 FOR | CTGCTTGCCACAGGTCTCC |
| TP53_Exon7 REV | AAGCCACAGGTTAAGAGGTCCC |
| TP53_Exon8 FOR | CTCCAGAAAGGACAAGGGTGG |
| TP53_Exon8 REV | GTTGGGCAGTGCTAGGAAAGAG |
| KRAS_Exon2 FOR | GTGTGACATGTTCTAATATAGTCA |
| KRAS_Exon2 REV | GAATGGTCCTGCACCAGTAA |
| KRAS_Exon3 FOR | TTGTTGAGTTGTATATAACACC |
| KRAS_Exon3 REV | ATTACTCCTTAATGTCAGCTTA |
| KRAS_Exon4 FOR | GTTACTAATGACTGTGCTATAAC |
| KRAS_Exon4 REV | ACTGATATATTAAATGACATAACA |
| BRAF V600E_FOR | ATGCTTGCTCTGATAGGAA |
| BRAF V600E_REV | GCATCTCAGGGCCAAA |
| NRAS_Exon2_FOR | ACCCTGATTACTGGTTTCCAAC |
| NRAS_Exon2_REV | ATGATCCGACAAGTGAGAGACA |
| NRAS_Exon3_FOR | TGCTTATTTAACCTTGGCAATAGC |
| NRAS_Exon3_REV | TGGTAACCTCATTTCCCCATAA |
| NRAS_Exon4_FOR | TTTTCTTATGTTCTGATAATATAT |
| NRAS_Exon4_REV | CATCTCTACCAGAGTTAATCAA |
| PI3K_Exon9_FOR | TTG CTT TTT CTG TAA ATC ATC TGT G |
| PI3K_Exon9_REV | CTG CTT TAT TTA TTC CAA TAG GTA TG |
| PI3K_Exon20_FOR | ACA TCA TTT GCT CCA AAC TGA |
| PI3K_Exon20_REV | CCT ATG CAA TCG GTC TTT GC |
